# Direct observation of the evolving metal–support interaction of individual cobalt nanoparticles at the titania and silica interface†

**DOI:** 10.1039/d0sc03113e

**Published:** 2020-10-26

**Authors:** Chengwu Qiu, Yaroslav Odarchenko, Qingwei Meng, Peixi Cong, Martin A. W. Schoen, Armin Kleibert, Thomas Forrest, Andrew M. Beale

**Affiliations:** Department of Chemistry, University College London 20 Gordon Street London WC1H 0AJ UK Andrew.Beale@ucl.ac.uk; Research Complex at Harwell (RCaH) Harwell Didcot Oxfordshire OX11 0FA UK; State Key Laboratory of Catalysis, Dalian Institute of Chemical Physics, Chinese Academy of Sciences Dalian 116023 China; Swiss Light Source, Paul Scherrer Institute Villigen 5232 Switzerland; Diamond Light Source Harwell Didcot Oxfordshire OX11 0DE UK

## Abstract

Understanding the metal–support interaction (MSI) is crucial to comprehend how the catalyst support affects performance and whether this interaction can be exploited in order to design new catalysts with enhanced properties. Spatially resolved soft X-ray absorption spectroscopy (XAS) in combination with Atomic Force Microscopy (AFM) and Scanning Helium Ion-Milling Microscopy (SHIM) has been applied to visualise and characterise the behaviour of individual cobalt nanoparticles (CoNPs) supported on two-dimensional substrates (SiO_*x*_Si(100) (*x* < 2) and rutile TiO_2_(110)) after undergoing reduction–oxidation–reduction (ROR). The behaviour of the Co species is observed to be strongly dependent on the type of support. For SiO_*x*_Si a weaker MSI between Co and the support allows a complete reduction of CoNPs although they migrate and agglomerate. In contrast, a stronger MSI of CoNPs on TiO_2_ leads to only a partial reduction under H_2_ at 773 K (as observed from Co L_3_-edge XAS data) due to enhanced TiO_2_ binding of surface-exposed cobalt. SHIM data revealed that the interaction of the CoNPs is so strong on TiO_2_, that they are seen to spread at and below the surface and even to migrate up to ∼40 nm away. These results allow us to better understand deactivation phenomena and additionally demonstrate a new understanding concerning the nature of the MSI for Co/TiO_2_ and suggest that there is scope for careful control of the post-synthetic thermal treatment for the tuning of this interaction and ultimately the catalytic performance.

## Abstract 2

Understanding the metal–support interaction (MSI) is crucial to comprehend how the catalyst support affects performance and whether this interaction can be exploited in order to design new catalysts with enhanced properties.

## Graphical abstract

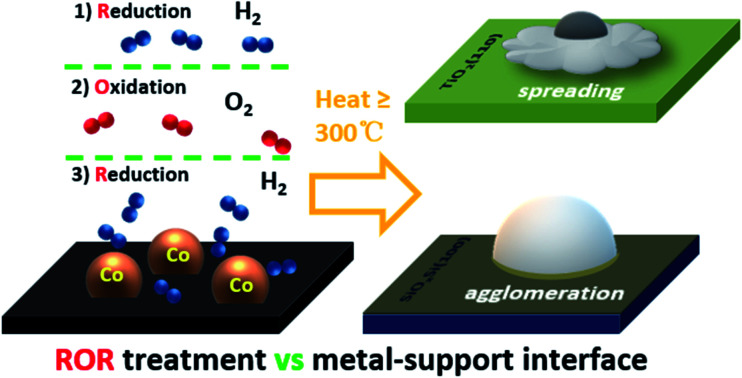

## Introduction

The nature and importance of the MSI at the interface between a metal nanoparticle and the support has been debated for a long time in the field of transition metal based heterogeneous catalysis.^1–4^ The MSI has been proposed to manifest itself in a variety of ways including: affecting charge transfer between metal nanoparticle and support, providing an interfacial perimeter where reactions can take place, allowing for the evolution in chemical composition at the perimeter (*i.e.* formation of solid solutions or alloys) and atom mobility (decoration or encapsulation, *etc.*).^5,6^ Indeed, such an interaction has been shown to be important for cobalt-based heterogeneous catalysts comprising metallic cobalt (Co^0^) deposited on a high specific surface area oxide support (*e.g.* TiO_2_, Al_2_O_3_, SiO_2_, *etc.*).^7–9^ In particular, for cobalt-based Fischer–Tropsch synthesis (FTS) catalysts, previous studies demonstrate that the MSI affects reducibility, stability and performance through structural transformation and migration of CoNPs.^10–12^ The significance of the MSI in cobalt-based catalysts is thought to primarily concern the interaction of oxides such as CoO with the support, as it was observed that Co_3_O_4_ present initially readily reduces to CoO but that the latter transformation from CoO to the Co metal polymorphs is difficult.^13^ Quite how the MSI effect manifests itself has been shown to depend somewhat on the reaction conditions.^14^ In the most basic sense, when CoNPs interact weakly with the support surface, migration occurs, leading to aggregation and a lowering of the cobalt dispersion and apparent reaction activity as the number of surface active sites decreases.^15,16^ Alternatively when the interaction between CoNPs and the support is intimate or strong they have been observed to become encapsulated by a TiO_*x*_ (*x* < 2) overlayer (∼few atomic layers thick) particularly after reduction.^9,16–18^ The encapsulation by TiO_*x*_ actually was also reported in other TiO_2_ supported metal NPs such as Au, Pt and Rh.^19–22^ Interestingly it has been reported that the amorphous TiO_*x*_ suboxide layer around CoNPs can be tuned and broken by sequential treatments in reducing/oxidising gases, resulting in significantly improved catalytic activity.^18^ More recent studies have proposed that cobalt atom migration can occur (on TiO_2_, SiO_2_ or Al_2_O_3_), leading to the formation of a Co-containing thin layer after H_2_ reduction or FTS reaction.^23–26^ Wolf *et al.* suggested that water plays an important role as far as inducing cobalt spreading on oxide supports (AlO_*x*_, SiO_*x*_ and TiO_*x*_) as well as the formation of non-reducible metal–support compounds during reduction and FTS.^27,28^ In many cases encapsulation, spreading or the formation of non-reducible metal–support compounds is inimical to catalyst performance as they lead to a reduction in the number of surface active sites by either decreasing the dispersion or else the extent to which they are reduced; this would likely be detrimental to activity if Co^0^ is the active phase as is generally thought. In comparison, as far as selectivity is concerned, it has been shown that the effect these changes have are minor and seemingly limited to shifting the hydrocarbon product distribution towards light hydrocarbons.^29,30^ To some extent, changing the temperature and gas atmosphere under which calcination and reduction occurs has been shown to mitigate these effects.^30–32^

Despite multiple studies, it is clear that the impact of the MSI effect on the nature, stability and performance of the Co NPs is not well understood.^5^ This is in part due to the fact that the majority of studies were performed on powders and pellets where the signal is typically averaged over a large number of, often, non-uniform CoNPs, meaning that insightful information may be difficult to decipher. Furthermore, when studying catalytic systems such as those used in FTS, high loadings (often up to 20 wt% Co) result in a number of particles in close proximity that can render the interrogation of the MSI signal difficult. Finally, since *ex situ* characterisation methods (*i.e.* where catalyst structure is interrogated before and/or after the reaction) are used in many of the studies, it is not always possible to identify which features of a spectrum, pattern or trace are pertinent to the MSI effect.

A combination of surface sensitive spectroscopic and microscopic methods with the single-particle resolution is required to explore the effect of the MSI in Co catalysts. To that end, we prepared two well-defined model 2D cobalt samples using flat single crystal SiO_*x*_Si(100) and TiO_2_(110) substrates as supports. The 2D samples contain a monolayer of highly monodispersed CoNPs with a large inter-particle distance (>100 nm) to eliminate the interaction between neighbouring nanoparticles and to overcome the limited spatial resolution of microscopy techniques. We used a combination of spectro-microscopy (particularly *quasi in situ* soft X-ray photoemission electron microscopy (X-PEEM) coupled with XAS) and AFM/SHIM microscopy as well as X-ray characterisation techniques including X-ray photoelectron spectroscopy (XPS) and grazing incidence X-ray scattering (GIXS) in order to obtain a consistent understanding of the behaviour of a supported CoNPs during reduction–oxidation–reduction (ROR) treatment; a common process used industrially to regenerate or enhance reaction activity of catalysts by improving metal dispersion, reducibility and MSI.^18,33–37^ Our results show in particular that CoNPs on Co/TiO_2_ have a tendency to spread on and embed into the TiO_2_ surface leading to CoNPs forming a fried-egg shape, whereas for Co/SiO_*x*_Si CoNPs are not stable and tend to agglomerate into bigger particles, more embedded into the surface support although possess a more ‘traditional’ hemispherical presentation at the support surface.

## Results and discussion

### Size and shape of the nanoparticles

**Fig. 1:**
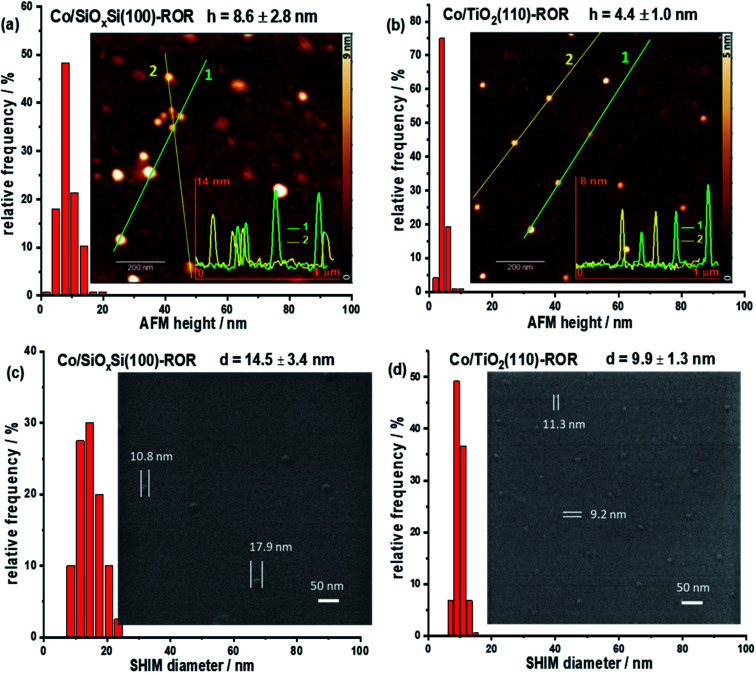
AFM (a and b) and SHIM (c and d) images and corresponding histograms of CoNPs supported on SiO_*x*_Si(100) (a and c) and TiO_2_(110) (b and d) substrates after ROR (623 K reduction) treatment. 1D profiles with the NPs height are shown on the inset in (a and b). Both height (*h*) and diameter (*d*) of CoNPs on SiO_*x*_Si(100) are larger than those on TiO_2_(110). For both samples the diameter is larger than the height indicating that NPs possess a semi-ellipsoidal shape.

AFM provides a simple and reliable way to measure a height as well as surface roughness of the NPs supported on a flat substrate. However, the length of the particle cannot be measured precisely due to the signal recorded in a lateral direction being convoluted with the dimension of the cantilever tip. SHIM by contrast, is a novel technique that can create a high resolution image of sufficient quality to determine the width of a particle. This allows for determining the morphology at the surface of the planar sample but, by being able to remove surface atomic layers, can also reveal what is beneath the surface, something that is not achievable with conventional electron microscopy.^38,39^ Thus, the 3D structure of supported nanoparticles can be determined by using these two methods in combination. Fig. 1 and S1† respectively contain the images acquired on freshly calcined samples and those that have undergone ROR and can be seen to contain isolated, hemispherical cobalt nanoparticles randomly distributed on SiO_*x*_Si and TiO_2_ substrates with an average inter-particle distance >100 nm. Specifically, the average height (*h*) and diameter (*d*) of CoNPs on the SiO_*x*_Si substrate in the calcined sample are determined to be 7.4 ± 3.1 nm and 9.3 ± 1.1 nm, respectively; while the corresponding dimensions of CoNP on TiO_2_ are 5.5 ± 2.2 nm and 7.8 ± 0.8 nm respectively. The nanoparticle size for the SiO_*x*_Si substrate is bigger than that for TiO_2_ in the calcined samples even though the same batch of nanoparticles (Fig. S2c and f†) was deposited on both substrates. The larger NP diameter with respect to its height indicates that the CoNPs on both substrates are semi-ellipsoidal.

After ROR treatment, the average height of CoNPs on SiO_*x*_Si increases by ∼16% to 8.6 ± 2.8 nm (Fig. 1a and S1a†) whereas on TiO_2_ the height decreases by ∼25% to 4.4 ± 1.0 nm (Fig. 1b and S1b†). On the other hand, the diameter of CoNPs on SiO_*x*_Si increases by 56% to 14.5 ± 3.4 nm (Fig. 1c and S1c†), and on TiO_2_ the CoNPs diameter increases by 27% to 9.9 ± 1.3 nm (Fig. 1d and S1d†). In addition, some particularly large CoNPs are observed on the Co/SiO_*x*_Si(100) sample, indicating movement (Fig. S5†) and agglomeration (Fig. 1a and S3†); this is in contrast to TiO_2_ where CoNPs broadly retain the large inter-particle distance appearing therefore to be more strongly bound to the surface (before ROR: 106.1 ± 39.5 nm and after ROR: 108.1 ± 33.9 nm according to the SHIM images).^26^

### Chemical state of the nanoparticles

*Ex situ* XPS Co 2p spectra (Fig. S4a†) contain a 2p_3/2_ peak (position ∼781 eV), Co 2p_3/2_–2p_1/2_ splitting energy (∼16 eV) and strong intensity of satellites at 787 eV and 803 eV and is consistent with the presence of Co^2+^ containing compounds (*i.e.* CoO, Co_2_SiO_4_ or CoTiO_3_) before and after ROR treatment in both Co/SiO_*x*_Si(100) and Co/TiO_2_(110) samples.^40^ A closer look at the Co 2p_3/2_ binding energy (BE) revealed slight differences for cobalt on TiO_2_(110) ∼781.0 eV *vs.* SiO_*x*_Si(100) ∼781.5 eV. Both BE values are higher compared to the reference CoO (780.6 eV) the origin of which has previously been proposed as evidence of a MSI effect.^41^ The absence of a peak at ∼457 eV (Fig. S4b†) indicates that no Ti^3+^ species are detected and that rutile TiO_2_ is not reduced during preparation/ROR treatment. It also implies that the formation of an amorphous suboxide (TiO_*x*_) overlayer encapsulating the supported NPs does not appear to have formed during treatment.^42^

**Fig. 2:**
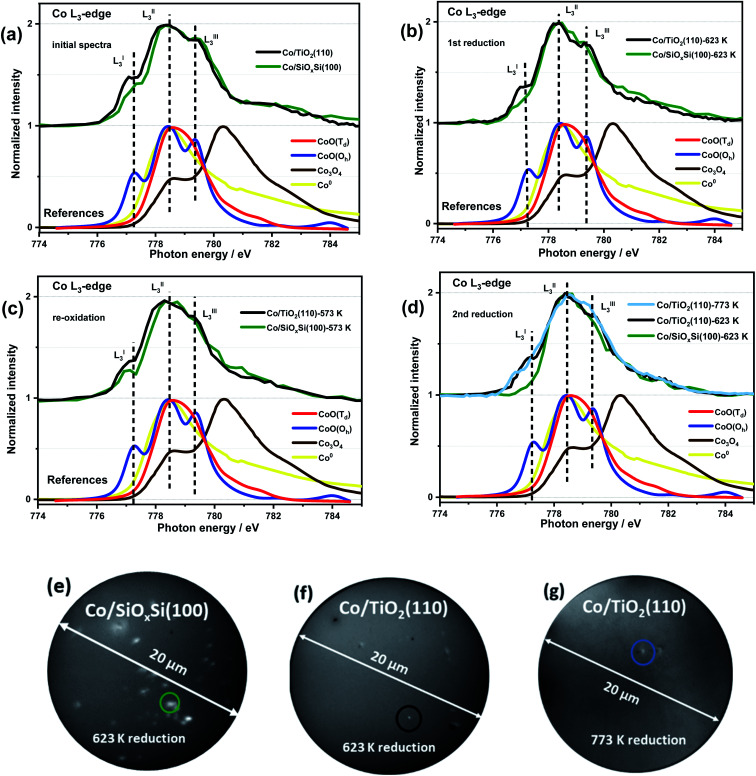
*Quasi in situ* XAS spectra of Co L_3_-edge recorded after each step of ROR process (a–d) and corresponding X-PEEM images (e–g) of circled CoNPs supported on SiO_*x*_Si(100) and TiO_2_(110) supports. X-PEEM field of view is equal to 20 μm. CoNPs on SiO_*x*_Si(100) are much easier to reduce and oxidise than those on TiO_2_(110); non-reduced CoNPs on TiO_2_(110) at reduction temperature as high as 773 K are observed.

In order to obtain more detailed, spatial and chemical insight into the nature of the CoNP species, *quasi in situ* soft XAS spectra and X-PEEM images for an individual CoNP after each step of ROR treatment were obtained and are shown in Fig. 2. The detailed description of the experiment setup used for X-PEEM analysis can be found in the ESI.† Three features are clearly identifiable in all spectra (indicated in Fig. 2a–d) and are assigned to L^I^_3_–L^III^_3_ which can be used to distinguish between the oxidation and local coordination state of Co-containing compounds.^43–46^ Notably, the presence of two prominent features (L^I^_3_ and L^III^_3_) in both samples indicates that Co is present primarily as high spin Co^2+^ species with octahedral (*O*_h_) coordination (*i.e.* CoO).^47,48^ For the XAS spectrum of the Co/SiO_*x*_Si(100) sample before the first reduction, shown in Fig. 2a (green line) and Fig. S8,† the presence of a strong main-feature at 778.4 eV (L^II^_3_) coupled with two weak shoulders at 777.3 eV (L^I^_3_) and 779.3 eV (L^III^_3_) suggests the formation of some (∼30% by linear combination analysis) tetrahedral (*T*_d_) Co^2+^ species (Fig. S8†), due to site substitution of *T*_d_ Si^4+^ in silica.^49^ No evidence for the spinel Co_3_O_4_ phase is found on either substrate.^45,47,50,51^

After reduction in H_2_, the Co/SiO_*x*_Si sample data exhibits a decrease in the intensities of L^I^_3_ and L^III^_3_ features, consistent with Co^2+^ reduction to Co^0^ metal, although the presence of a distinct L^I^_3_ feature (dark green trace in Fig. 2b) suggests reduction is not complete. For the Co/TiO_2_ the L^I^_3_ and L^III^_3_ components also decrease however, a comparatively strong (∼25% more CoO than in Co/SiO_*x*_Si sample) L^I^_3_ feature indicates that a significant portion of Co^2+^ remains suggesting that it is more difficult to reduce. Re-oxidation sees the original Co^2+^-containing spectrum largely restored for Co/SiO_*x*_Si (Fig. 2c), although in this new spectrum the L^III^_3_ feature (a multiplet from octahedral Co^2+^ in CoO) is diminished in comparison to the initial XAS profile in Fig. 2a. In comparison, only a minor re-oxidation occurs in Co/TiO_2_(110) as evidenced by a small increase in normalized intensity (NI) of the L^I^_3_ feature (ΔNI of L^I^_3_ ≈ 0.06) in Fig. 2c. The L^III^_3_ feature is not clearly detected either, indicating that the Co^2+^ is partially distorted,^52^ or the oxidation (for both samples O_2_ partial pressure was 5 × 10^−7^ mbar at 573 K) is weak.

After the second reduction, the disappearance of L^I^_3_ and L^III^_3_ features in Co/SiO_*x*_Si indicates a complete NP reduction (*i.e.* the spectrum resembles a Co^0^ spectrum, Fig. S9a†). Thus the Co^2+^ species with *T*_d_ coordination initially present (likely as cobalt silicate Co_2_SiO_4_) does not appear to affect the reducibility of cobalt in the sample greatly.^33^ In comparison, for Co/TiO_2_, the intensities of L^I^_3_ and L^III^_3_ multiplets again decrease back to the same intensity as observed during the first reduction (Fig. 2d). Increasing the reduction temperature from 623 K to 773 K leads to only a minor further reduction (shown in Fig. 2d light blue line, ΔNI of L^I^_3_ ≈ 0.14 lower than the value obtained at 623 K) consistent with a particularly strong Co–TiO_2_ support interaction.

### Spreading of Co on TiO_2_

**Fig. 3:**
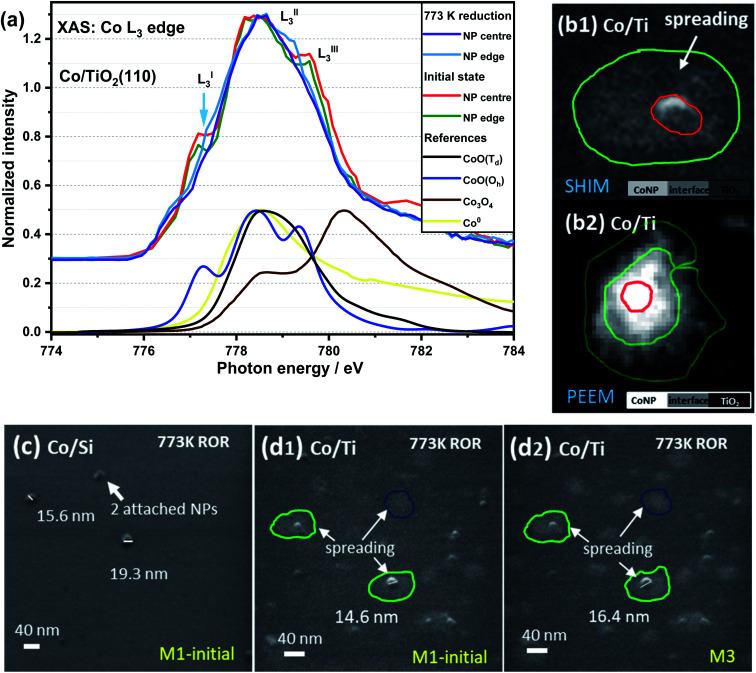
XAS spectra of Co L_3_-edge in the edge and centre of a TiO_2_ supported CoNP (a), and SHIM images of Co/SiO_*x*_Si(100) (c) and Co/TiO_2_(110) (b1, d1 and d2) sample after 773 K ROR treatment. M1 and M3 stand for 1 and 3 times that helium ion milling was employed to remove a surface layer of the sample (typical depth: sub-nm to μm, depending on beam intensity, dwell time and sample properties^53,54^), *i.e.* CoNPs on SiO_*x*_Si ∼0.2 nm per mill and on TiO_2_ ∼0.25 nm per mill. The feint grey patches (circled) shown in (b1, d1 and d2) highlight the spreading of Co on TiO_2_ (up to ∼40 nm away from the NPs); the sample cannot be fully reduced as shown in the XAS spectra in panel (a). PEEM image (b2) was collected at the Co edge (778.5 eV).

Fig. 3a contains Co L_3_-edge soft XAS spectra from the centre and the edge of an individual CoNP on Co/TiO_2_(110). The corresponding X-PEEM image is shown in Fig. 3(b2) and can be compared with a typical SHIM image after 773 K ROR treatment (Fig. 3c and d). In the pristine sample, the L^I^_3_ feature at the edge of NP has a lower intensity than the signal recorded in the centre of the NP, indicating that whilst the spectral features are consistent with the presence of *O*_h_ Co^2+^ throughout the particle, the perimeter contains an additional component to CoO indicating the cobalt environment at the metal–support interface is different to that of the centre of NP.^55^ After 773 K reduction, the position of the L^I^_3_ feature at the metal–support interface as well as in the centre of NP decreases from 777.3 to 776.8 eV with a slightly lower intensity than that seen at the edge (ΔNI of L^I^_3_ ≈ 0.09). This further demonstrates that cobalt at the NP edge has a stronger interaction with the TiO_2_ support, thereby making it more difficult to reduce. The presence of oxidised Co at the edge of the NPs in the X-PEEM images is consistent with the spreading of cobalt observed in SHIM images *vide infra*.

In order to correlate spatially resolved soft X-ray spectroscopy data with higher resolution microscopy SHIM measurements (Fig. 3c, d and S3†) were performed. The SHIM contrast signal shows white dots corresponding to the metal-containing nanoparticles against the grey background (substrate) in both samples. Interestingly, there is a loss of the initial semi-ellipsoidal shape of the TiO_2_ supported NPs (Fig. S3†) accompanied by evidence of diffuse ‘grey patches‘ with up to ∼40 nm diameter around the NPs. We attribute this to spreading of Co onto the TiO_2_ substrate surface. This (grey patch) is seen with or without a co-located CoNP (see green and blue circles in Fig. 3d). This phenomenon also occurs during ROR at 623 K although the spreading observed in SHIM as shown in Fig. S3b,† inset occurs to a lesser extent. During systematic He ion beam milling to remove constituent atoms a layer at a time, the diameter of CoNPs supported on TiO_2_ and on SiO_*x*_Si seems to ‘increase’ (Fig. S3†) confirming the previous assignment of the CoNPs' shape. After milling 3 times, the patches on TiO_2_ (after ROR at 773 K) also become bigger and the contrast between the patches and the substrate is more noticeable, making the spreading more visible. After milling 30 times (Fig. S3†), all CoNPs on SiO_*x*_Si substrate can still be observed (they appear more disc-shaped) but on the TiO_2_ support many of NPs and the surrounding diffuse patches are removed suggesting a limit to the penetration of Co below the TiO_2_ surface. A quick estimation reveals the average depth of cobalt ingress is ∼3 nm as the diffuse grey patches are typically removed after 12 milling events. Assuming an intimate contact at the interface allows us to propose a ‘fried-egg like’ shape when understanding the effect of the interaction of CoNPs at the TiO_2_ surface although the Co is observed to penetrate quite some way down.

The effect of the support on the CoNPs structure after reduction is also examined by employing surface X-ray scattering that can be effectively used to monitor the structural changes of the supported metal nanoparticles.^56^ The grazing incidence X-ray diffraction (GIXD) data for Co/SiO_*x*_Si(100) and Co/TiO_2_(110) samples after reduction are presented in Fig. S11† showing the difference in crystalline structure of the CoNPs on the two supports. The surface X-ray diffraction pattern for Co/SiO_*x*_Si sample after reduction shows the presence of reflections at *s*_*z*_ = 0.48 and 0.56 Å^−1^ that can be indexed as the fcc phase of metallic cobalt. In contrast the signal from metallic cobalt is barely observable for Co/TiO_2_. Corresponding grazing incidence small-angle X-ray scattering (GISAXS) data however, reveal the presence of well-defined nanoparticles (Fig. S12†) in both samples. The average diameter of Co particle on the SiO_*x*_Si substrate after reduction is determined to be ∼6.8 nm, whereas the average height only ∼4.7 nm both values of which are comparable to that seen by SHIM and AFM data after ROR treatment. We note that GISAXS data on CoNPs were recorded after *in situ* reduction whilst the AFM and SHIM are recorded on samples after ROR treatment exposed to the atmosphere and will therefore contain an oxide (CoO) overlayer and more agglomeration. In contrast, the average values obtained from fitting the GISAXS data for CoNPs supported on TiO_2_ suggests a highly asymmetric structure with a diameter of 16.47 nm and height of 4.15 nm (Fig. S12†). Again, notwithstanding the small differences in height and width, the GISAXS data captures the same CoNP asymmetry that was previously observed with AFM & SHIM. Comparing between samples it can be summarised that from the GISAXS data the average lateral diameter of the particles on Co/TiO_2_ are four times larger than they are high and confirm the presence of a comparatively spread CoNP than that seen on the SiO_*x*_Si substrate where the particles appear more semi-ellipsoidal (Fig. S12e†).

## Discussion

**Scheme 1:**
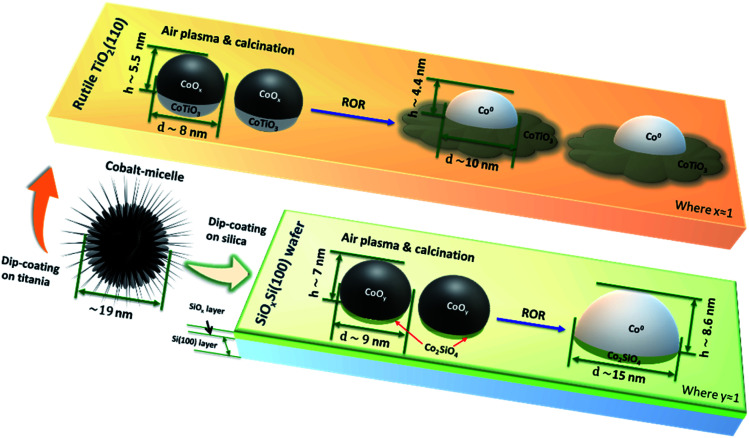
Schematic of the Co nanoparticles evolution on TiO_2_(110) and SiO_*x*_Si(100) substrates after ROR. The spreading of CoNPs onto the surface of TiO_2_ forms a fried-egg shape resulting in strong interaction with the support to form CoTiO_3_, while CoNPs on SiO_*x*_Si tend to move and agglomerate into bigger particles.

During the ROR treatment CoNPs spread onto and also partially embed into TiO_2_(110) substrate more strongly than into SiO_*x*_Si(100). This is confirmed by the lower AFM height and smaller SHIM diameter (excluding the spreading cobalt, Scheme 1) of CoNPs on TiO_2_ as well as by the grey patches surrounding CoNPs in electron microscopy images after SHIM (Fig. 3b and S3 inset†). We observe that the Tamman and Hüttig temperatures (at which lattice and surface atoms becomes significantly mobile, respectively) of bulk CoO (mp 2068 K) are 1034 K and 620 K, respectively;^57^ while for metallic cobalt (mp 1768 K) *T*_T_ = 884 K and *T*_H_ = 530 K.^58,59^ Tamman and Hüttig temperatures for our Co NPs (mainly CoO initially and after re-oxidation, and Co^0^ during the two reduction steps) will be lower than the above values^60,61^ (*e.g.*, ∼10% lower for 5 nm NP^61^), particularly considering the small size of CoNPs on TiO_2_ (Fig. 1, S1 and S2†). Therefore the ROR treatment (reduction at 623 K or 773 K, oxidation at 573 K) enables Co atoms at the NP surface to be mobile irrespective of whether the NP is reduced to metallic cobalt or remains oxidised (CoO phase). Note however, that NP mobility is also observed in samples undergoing ROR at lower *T* (623 K) albeit to a lesser extent.

The higher temperatures encountered in the reduction steps renders this process more significant as far as Co migration to the titania substrate is concerned while we propose that re-oxidation is probably responsible for the ‘anchoring’ of the migrated Co by dint of the formation of a Co–TiO_2_ (CoTiO_3_) support interaction similar to what is reported for Co/SiO_2_ catalysts.^37^ We observe that reported free energy of formation values from oxides of Co_2_SiO_4_ (−12.03 kJ mol^−1^ (ref. 62)) is higher than that of CoTiO_3_ (−18.41 kJ mol^−1^ (ref. 63)) suggesting that cobalt titanate (*O*_h_) forms on Co/TiO_2_ more readily. The unreduced Co^2+^ species determined by the significant L^I^_3_ feature in the Fig. 2b and d after first and second reduction (black line) are considered to be pertinent to this interface compound. The cobalt spreading might be independent of TiO_2_ polymorph; indeed recent studies have reported that Co may be more mobile across more traditional catalysts that comprise both anatase (majority) and rutile polymorphs.^24^ Whereas promoter (*e.g.* Mn) addition seems to protect the TiO_2_ supported CoNPs and to inhibit the spreading of cobalt *via* Co_*x*_Mn_1−*x*_O compound formation during reduction.^64–66^ We do not clearly observe the formation of an amorphous TiO_*x*_ (*x* < 2) overlayer (no energy shift in Fig. S7†) during reduction and which has been reported as a major contributor to the deactivation of cobalt catalysts in literature.^8,17^ Our results, in conjunction with these past studies, suggest that for CoNPs on *crystalline* TiO_2_, the formation of this overlayer is not a main cause of deactivation. The question then remains as to whether this leads to a more or less active catalyst? As indicated in this study and alluded in others, in FTS a high degree of re-dispersion of Co across a support can render it more difficult to reduce and therefore less active and selective and so in this sense it is a process that potentially leads to CoNP inactivity; for example it has been reported that CO conversion declines although the product selectivity remains largely unaffected.^18^ This migration and spreading of CoNPs either as oxidic or carbidic species has also been implicated in deactivation of Co FTS catalysts. The presence of carbon deposits in re-dispersed CoNPs has also been seen in reacted samples and been associated with deactivation possibly *via* the formation of either cobalt carbide and/or coke.^24^ On the other hand, previous observations of the presence of a CoO_*x*_–titania interfacial-compound and in particular the presence of oxidic Co, has been shown to result in a more active catalyst (albeit with a higher tendency to produce alkanes) for both CO and CO_2_ hydrogenation than those where TiO_*x*_ has been proposed to encapsulate CoNPs.^67^ In addition, a recent study by de Jong and co-workers^18^ showed that the ROR treatment of a Co/TiO_2_ catalyst led to improved catalytic activity, which they attributed to a change in the metal–support interaction (based on H_2_ uptake). However, in both of these two recent studies, the authors were not able to rationalise fully how the gas treatments affect the CoNP′ structure. The various characterisation techniques employed here allow us to illustrate in Scheme 1, how spreading of Co on TiO_2_ results in the production of a fried-egg-shape on the support surface leading to CoNPs with a greater surface area (*i.e.* for H_2_ uptake and catalytic activity) than a spherical or hemispherical structure seen (postulated) in the wider literature for CoNPs. This structure would lead to a greater degree of oxidised Co previously observed although the true impact that this has on catalytic performance is not possible from these samples due to the low quantity of metal present.

In contrast, the shape of the CoNPs on SiO_*x*_Si appear more typically semi-elliptical, due to the weaker MSI and which also results in more significant metal aggregation with time/treatment.^68^ The aggregation of CoNPs supported on SiO_2_-support samples has previously been seen and seems related to the difficulty in forming stable mixed-oxide interfacial compounds (*i.e.* Co_2_SiO_4_ requiring at least more Co to be present than support oxide), when compared to Co/TiO_2_. Severe aggregation has previously been reported to adversely affect catalytic performance resulting in low activity and poor C_5_^+^ selectivity and stability.^69–71^ We note that the interfacial compounds are still present in the CoNPs (*i.e.* a small L^I^_3_ feature in the Co L_3_-edge XAS data shown in Fig. S10a† can be clearly seen in the spectra at the edge of the CoNP while it is absent from the spectra recorded in the centre of the particle) after ROR treatment. However, the strong correlation of FTS performance and the presence of Co^0^ when supported on SiO_2_ suggests that the interfacial compound and oxidised Co^2+^ should be avoided where possible. We note that our substrate is comprised of a surface layer of SiO_2_, below which Si exists in a lower oxidation state (Si^*n*+^, *n* < 4), thus potentially leading to weaker interaction when compared to CoNPs located on a more conventional SiO_2_ support. However since there is ∼4 nm of SiO_*x*_ (estimated from Si 2p spectra in Fig. S13b†),^72^ the effect of the subsurface Si layer is not expected to be significant. An illustration of the determined interaction of the CoNPs with the SiO_*x*_ layers is shown in both Scheme 1 and Fig. S13a.†

## Summary and conclusion

The goal of this study is to better understand the effect of a catalyst support on the shape and stability of Co NPs during the various stages of ROR. To this end a polymer inverse micelle encapsulation method has been used to produce an ordered array of CoNPs on 2D single crystals of silica and titania. A combination of AFM, X-PEEM, soft X-ray XAS, SHIM and GIXS have been applied, which allow us for the first time directly visualise and interrogate the interfaces of individual nanoparticles with the substrate. A stronger metal–support interaction between CoNPs on TiO_2_(110) than for CoNPs on SiO_*x*_Si leads to the retention of large inter-particle distances although a significant change in morphology eventually leading the CoNPs to adopt a fried-egg-like shape. On the one hand, this spreading increases the surface area of the CoNPs and their overall electronic state, both of which may prove beneficial for reactivity. But on the other hand and particularly if the spreading becomes extensive, it manifests in more non-reducible CoTiO_3_, which is likely to lead to a decrease in catalytic activity for reactions like FTS and in other reactions where metal cobalt is thought to be the active phase. In contrast, CoNPs supported on SiO_*x*_Si are not as stable, and undergo significant aggregation. The mobility of CoNPs on silica is clearly detrimental to catalyst performance and suggests that at least ROR procedures utilizing silica substrates will have to be performed at lower temperatures compared to TiO_2_ supported catalysts. Working at lower temperatures will of course have an adverse effect on intrinsic Co mobility and may leave only a limited temperature window for optimising CoNP nanostructures on silica. Our results show that there is scope for further optimisation/tuning of the CoNPs/TiO_2_ interface to achieve better performance; particularly the application of heat and the nature of the reactive gas atmosphere and possibly in combination with a targeted initial particle size. They also indicate that there is a definitive place for the study of 2D catalysts, particularly where the probing of the fundamentals of catalysis is concerned. Beyond what we have shown here regarding the NP–support interface, 2D catalysts also allow for studying phenomena such as shape/size evolution under reaction conditions and in particular, when combined with preparation methods for controlling particle size and *in situ* nano/micro-spectroscopies it is possible even to interrogate the behaviour of individual particles under reaction conditions. This last aspect could prove very powerful in unravelling the effect of particle size on catalytic activity.

## Conflicts of interest

There are no conflicts to declare.

## Supplementary Material

SC-011-D0SC03113E-s001
