# Supplementary material for: Direct observation of the evolving metal–support interaction of individual cobalt nanoparticles at the titania and silica interface
Source: Chem Sci. 2020 Oct 26;11(48):13060–70. doi: 10.1039/d0sc03113e (PMC8163327; doi:10.1039/d0sc03113e)
Supplement: SC-011-D0SC03113E-s001 [file SC-011-D0SC03113E-s001.pdf]

Electronic Supplementary Material (ESI) for Chemical Science.
This journal is © The Royal Society of Chemistry 2020

Electronic Supporting Information

Direct observation of the evolving metal-support interaction of individual cobalt nanoparticles at the titania and silica interface

Chengwu Qiu[1,2], Yaroslav Odarchenko[1,2,†], Qingwei Meng[3], Peixi Cong[1,2], Martin A.W. Schoen[4], Armin Kleibert[4], Thomas Forrest[5], Andrew M. Beale[1,2,*]

1 Department of Chemistry, University College London, 20 Gordon Street, London, WC1H 0AJ, UK

2 Research Complex at Harwell (RCaH), Harwell, Didcot, Oxfordshire, OX11 0FA, UK

3 State Key Laboratory of Catalysis, Dalian Institute of Chemical Physics, Chinese Academy of Sciences, Dalian, 116023, China

4 Swiss Light Source, Paul Scherrer Institute, Villigen, 5232, Switzerland

5 Diamond Light Source, Harwell, Didcot, Oxfordshire, OX11 0DE, UK

**corresponding author: Andrew.Beale@ucl.ac.uk*

†Current address: Finden Limited, Merchant House, 5 East St Helens Street, Abingdon OX14 5EG, UK

Table of contents

1. Sample preparation.
2. AFM.
Figure S1. AFM images and corresponding histograms of CoNPs supported on $SiO_xSi(100)$ and $TiO_2(110)$.
3. SHIM and TEM.
Figure S2. SHIM and TEM images and corresponding histograms of CoNPs supported on $SiO_xSi(100)$ and $TiO_2(110)$ before ROR treatment.
Figure S3. SHIM images of $Co/SiO_xSi(100)$ and $Co/TiO_2(110)$ after ROR and 773 K ROR treatment with helium ion milling; and after 773 K R and 773 K RO treatment.
4. XPS.
Figure S4. Co 2p, Ti 2p and O 1s XPS spectra of catalysts $Co/SiO_xSi(100)$ and $Co/TiO_2(110)$ before and after ROR treatment, and Co 2p spectra of the catalysts without calcination.
5. X-PEEM.
Figure S5. X-PEEM images of observed movement of CoNPs supported on $SiO_xSi(100)$ during ROR process.
Figure S6. X-PEEM images of selected regions in the process of ROR.
Figure S7. XAS spectra of Ti $L_{3,2}$-edge on $Co/TiO_2(110)$ before and after ROR process.
Figure S8. Comparison of Co L_3-edge XAS spectra between initial state of a CoNP on $TiO_2(110)$ and $SiO_xSi(110)$, and standard T_d, O_h Co^{2+} XAS spectra.
Figure S9. XAS spectra of Co L_3-edge of CoNPs supported on $SiO_xSi(100)$ and $TiO_2(110)$ at different steps of ROR process.
Figure S10. Comparison of XAS spectra of Co-L_3-edge in the centre and edge of a CoNP in $Co/SiO_xSi(100)$ before and after ROR treatment.
6. Surface X-ray Scattering
Figure S11. 1D GIXD profiles of $Co/SiO_xSi(100)$ and $Co/TiO_2(110)$ catalysts.
Figure S12. Experimental vs. fitted 2D GISAXS images of reduced $Co/SiO_xSi(100)$ and $Co/TiO_2(110)$ catalysts.
Figure S13. Schematic of the SiO_x layer in Si(100) substrate and interaction of CoNPs with SiO_xSi substrate.

1. Sample preparation

Cobalt nanoparticles (CoNPs) were synthesized by using reverse micelles comprising polystyrene block poly 2-vinylpyridine (PS-b-P2VP) diblock copolymer (PS327000-b-P2VP70000, Polymer Source Inc.) and $Co(NO_3)_2{\cdot}6H_2O$ (Sigma-Aldrich, ≥ 98%) under room temperature (293 K) and atmospheric conditions. The obtained cobalt nitrate micelles were coated onto a flat substrate ($SiO_xSi(100)$ or rutile $TiO_2(110)$) by dipping into the solution, followed by removing the substrate at a constant velocity of 5 mm/min. This procedure resulted in a monomicellar film on the substrates' surface with a high degree of hexagonal order. The polymer molecular coat on the cobalt salt was removed by air plasma (100 W, 0.5 mbar, 15 min). The details of synthesis method can be found elsewhere.[1,2] Then the prepared samples were heated to 773 K and kept for 6 h in air for thermal treatment before ROR. Due to spatial resolution restraints of X-PEEM, the samples for X-PEEM measurement were diluted with an average interparticle distance of >100 nm to avoid the overlapping of different particle signals.[3] After calcination, the samples were treated by Reduction-Oxidation-Reduction (ROR) process: reduction (H_2 $1x10^{-6}$ mbar, 623 K, 1h), oxidation (O_2 $5x10^{-7}$ mbar, 573 K, 1h) and the second reduction (H_2 $1x10^{-6}$ mbar, 623 K, 1h). The reduction treatment is denoted as 'R' while oxidation treatment as 'O'. For $Co/TiO_2(110)$, however, cobalt was not fully reduced at above reduction temperature. Thus, after the normal ROR treatment, the sample was reduced at 773 K, oxidised at 573 K and reduced again at 773

K, named 773 K ROR in the manuscript. For comparison, the Co/SiO_xSi(100) sample was also subjected to 773 K ROR.

2. AFM

The supported NPs after air plasma etching, calcination and ROR treatment were measured in air by Bruker Veeco MultiMode V atomic force microscope (AFM) at the Diamond Light Source (DLS) in a tapping mode and a scan rate of 2 Hz (293 K, 1 atm; cantilever Bruker RTESPA-300). From the obtained AFM images the CoNPs size (height) and distribution were analysed by Gwyddion 2.49 software.[4,5]

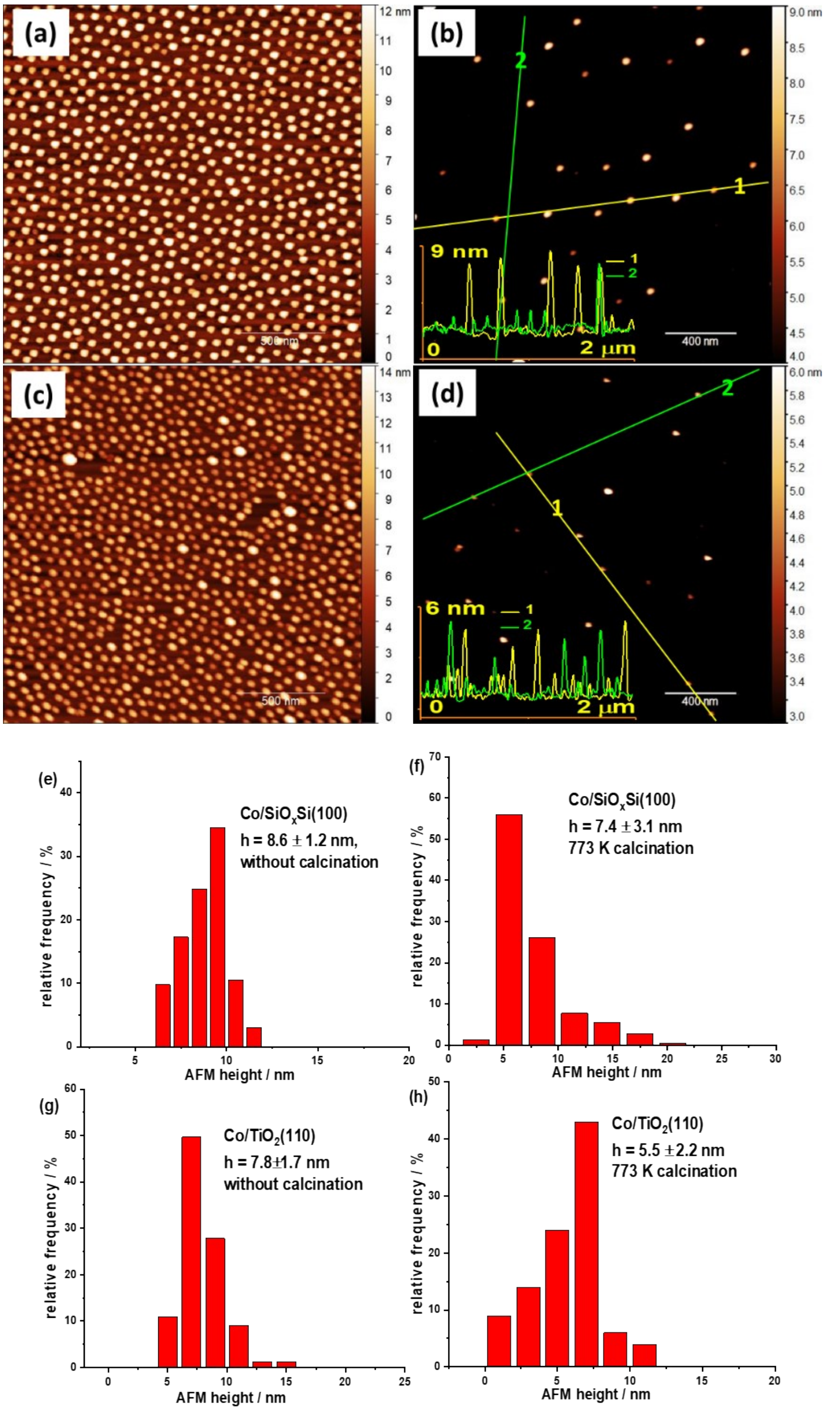

Figure S1. AFM images (a-d) and corresponding histograms (e-h) of CoNPs supported on SiO_xSi(100) and TiO_2(110) substrates. (a, b) AFM images are Co/SiO_xSi(100) and (c, d) AFM images are Co/TiO_2(110) catalysts. (a, c) AFM images corresponds to the catalysts without calcination and dilution, (b, d) images are 60-fold diluted samples before ROR treatment. The CoNPs with similar size (around

8.0 nm in height) before calcination are dispersed on both substrates $SiO_xSi(100)$ and $TiO_2(110)$ uniformly. The CoNPs on the two substrates used for X-PEEM analysis disperse well but with different sizes (S1b&d). CoNPs on $SiO_xSi(100)$ substrate (7.4±3.1 nm in average, S1f) seem agglomerated with some significantly bigger NPs (2-3 fold) observed; while that on $TiO_2(110)$ show opposite results, CoNPs size become much smaller after calcination (5.5±2.2 nm, S1h) for $TiO_2(110)$ substrate.

3. SHIM and TEM

The NP size and distribution of samples before and after ROR were measured by Carl Zeiss Orion NanoFab Scanning Helium Ion Microscope (SHIM, tilt angle = 45°, GFIS acceleration V = 25 kV) at the London Centre for Nanotechnology (LCN) in University College London.

Around 10 drops of polymer micelles solution were deposited onto a gold TEM grid (mesh size 200) with a carbon film, dried at room temperature in air and measured by a JEM2100 TEM 200 kV instrument.

The SHIM and TEM data were analysed by using the ImageJ 1.52e software.[6,7]

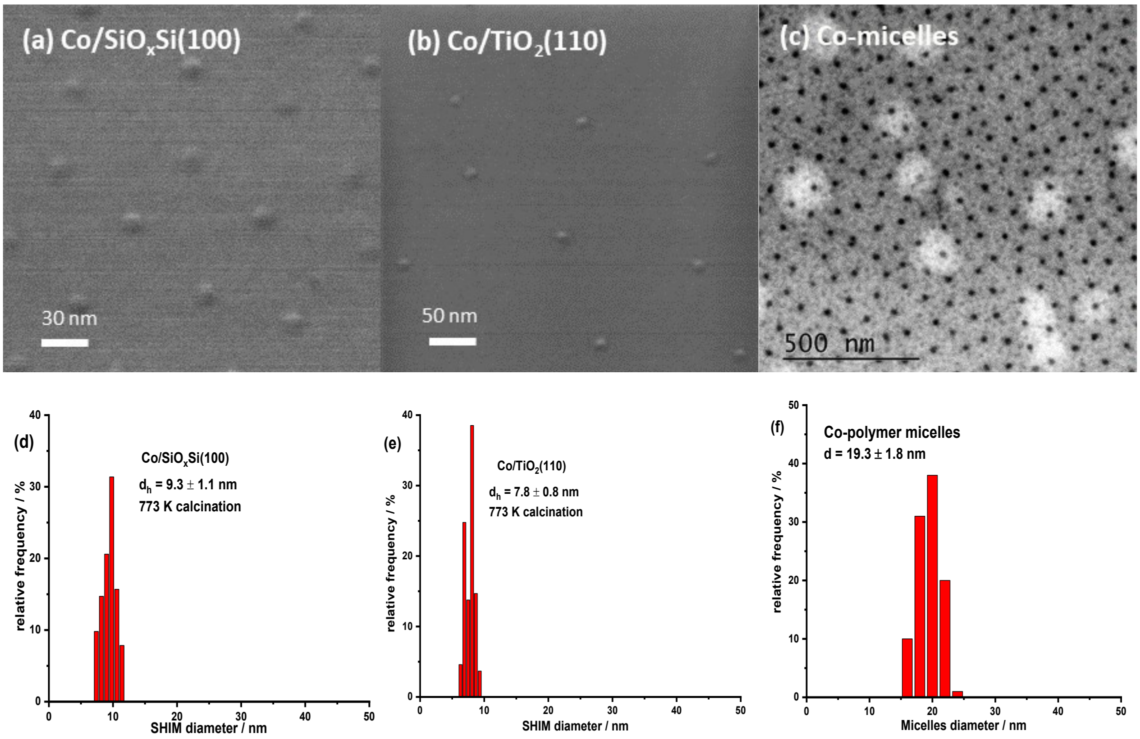

Figure S2 SHIM (a, b) and TEM (c) images and corresponding histograms (d-f) of CoNPs supported on $SiO_xSi(100)$ and $TiO_2(110)$ before ROR treatment. The displayed horizontal CoNP size on SiO_xSi (9.3±1.1 nm) is bigger that on TiO_2 substrate (7.8±0.8 nm), which is well in accordance with the AFM vertical size. As the cobalt-micelles (19.3±1.8 nm, Figure S2c&f) used for samples preparation are same and all the other treatment conditions (including dip-coating, plasma and calcination) are also consistent. The size differences in the two catalysts are only due to the support effects.

(I)

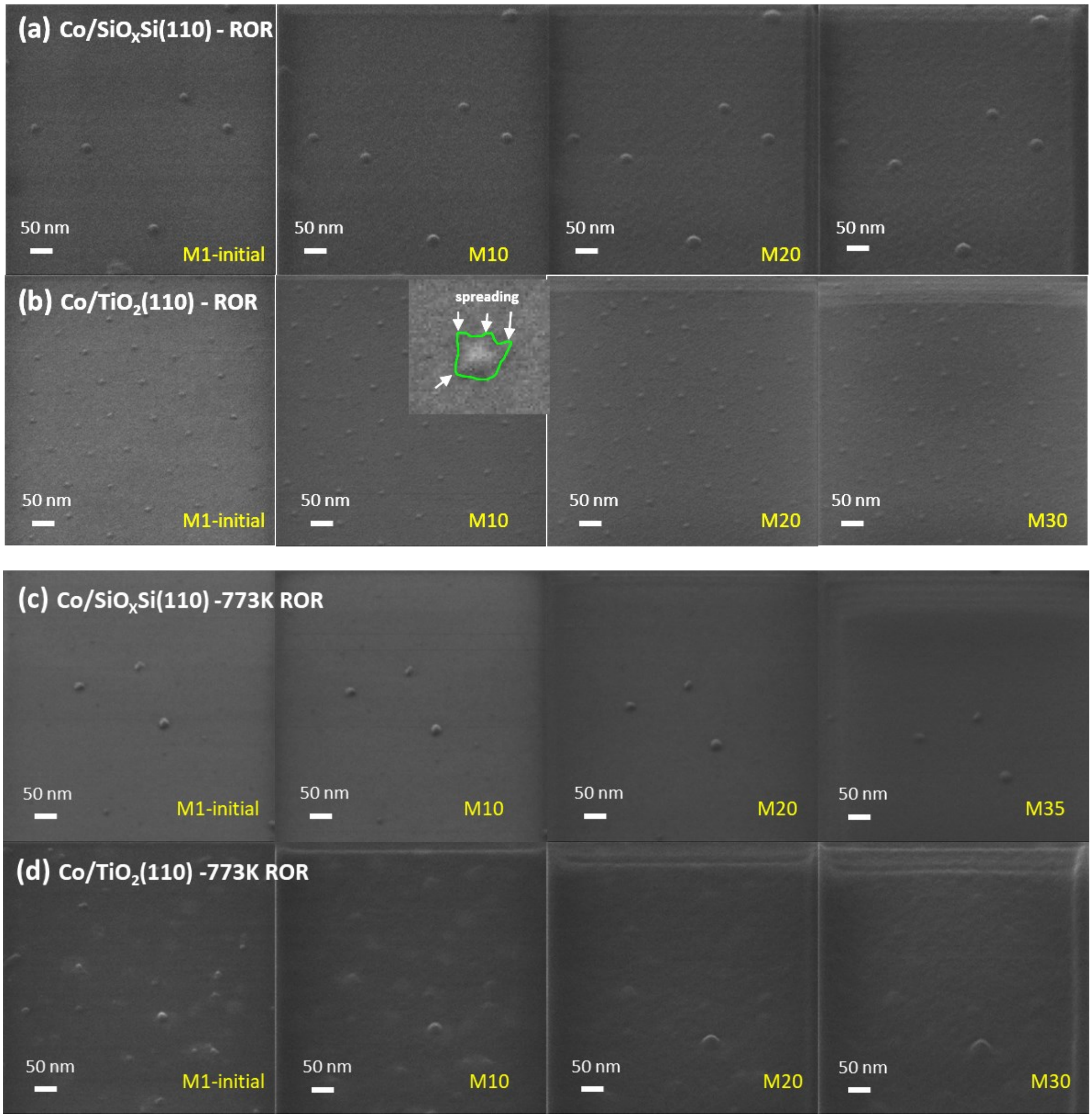

(II)

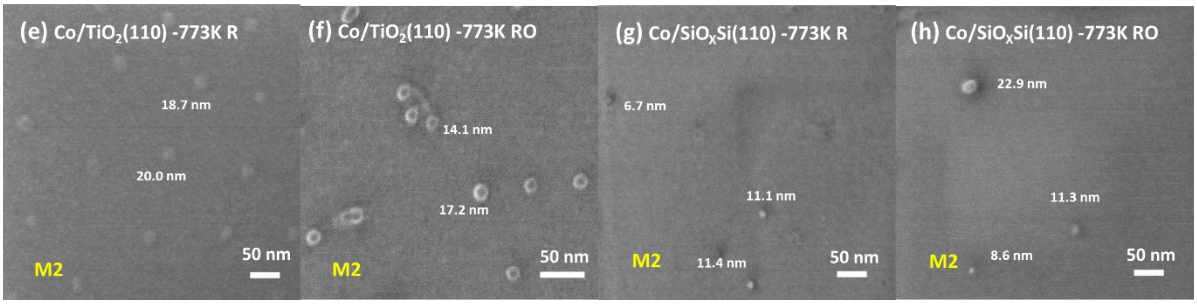

Figure S3. (I) SHIM images of Co/SiO$_x$Si(100) (a, c) and Co/TiO$_2$(110) (b, d) after ROR (a, b) and 773 K ROR (c, d). Treatment with helium ion milling for 1, 2, 10, 20 and 30 times denoted as M1, M2, M10 and M20, respectively. NPs diameters become larger with ion milling, meaning the embedment is happened in both catalysts. (II) SHIM images of Co/TiO$_2$(110) (e, f) and Co/SiO$_x$Si(100) (g, h) after 773 K R (e, g) and 773 K RO (f, h) treatment.

4. XPS

X-ray photoelectron spectroscopy was performed on a Thermo Fisher Scientific NEXSA spectrometer at HarwellXPS. Samples were analysed using a micro-focused monochromatic Al X-ray source (72 W) over an area of approximately 400 microns. Data were recorded at pass energies of 200 eV for survey scans and 50 eV for high resolution scan with 1 eV and 0.1 eV step sizes, respectively. Charge neutralisation of the sample was achieved using a combination of both low energy electrons and argon ions. All the samples were measured under the vacuum of 10^{-9} mbar and room temperature. The obtained data were analysed by CasaXPS (Version 2.3.19PR1.0).[8] The binding energy of Co 2p, O 1s, Ti 2p and Si 2p was calibrated by using C 1s (284.8 eV).

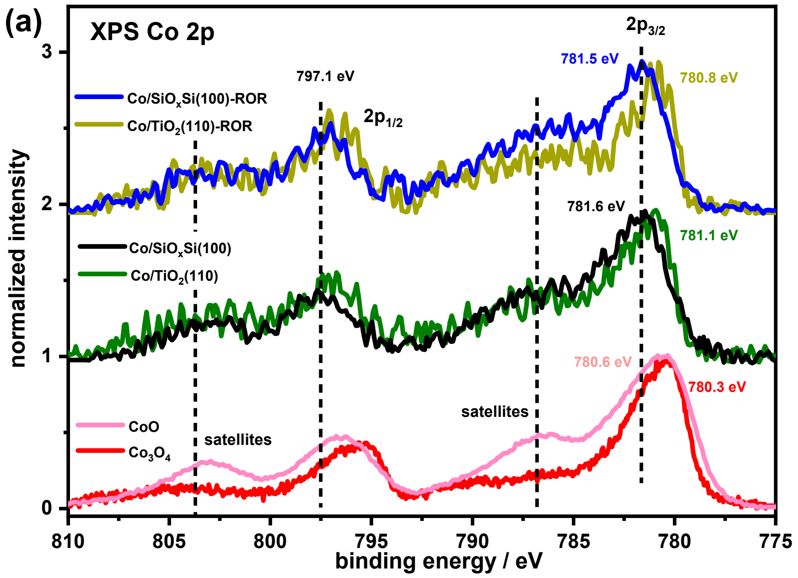

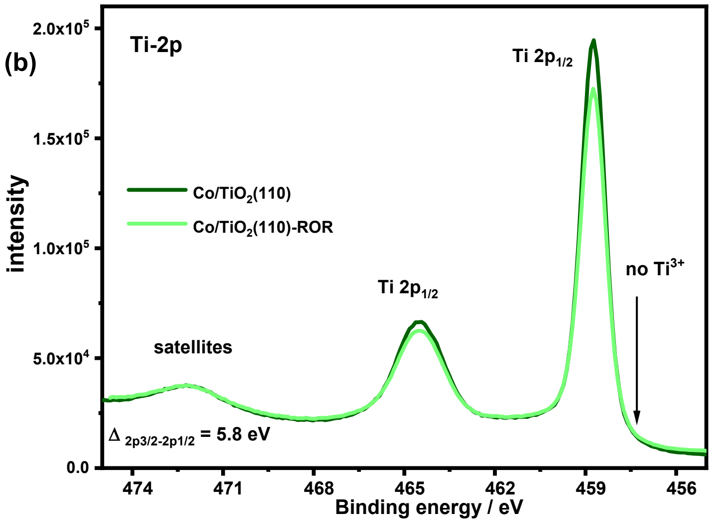

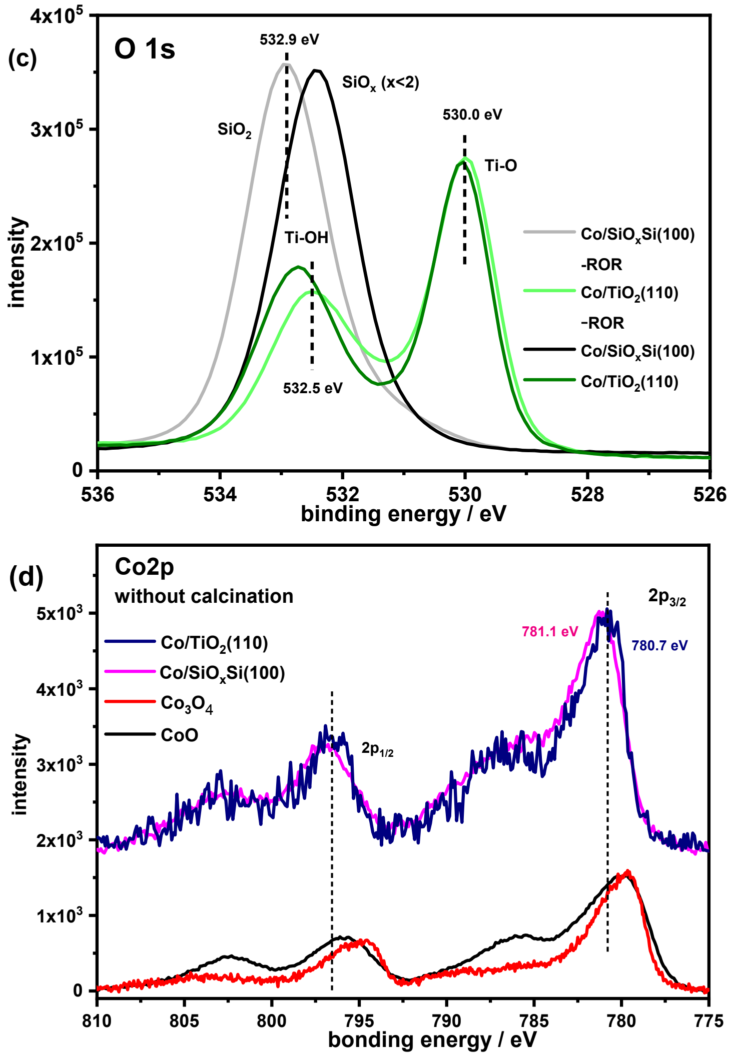

Figure S4. Co 2p (a), Ti 2p (b) and O 1s (c) XPS spectra of catalysts Co/SiO_xSi(100) and Co/TiO_2(110) before and after ROR treatment; (d) Co 2p spectra of the catalysts without calcination showing as CoO.

5. X-PEEM

X-ray spectroscopy and microscopy were simultaneously measured out at the Surface/Interface: Microscopy (SIM) beamline at the Swiss Light Source (SLS) and I06 beamline at DLS using an X-PEEM equipped with an energy analyser. The catalysts employed were Co/SiO_xSi (100) and Co/TiO_2(110) with same initial cobalt-polymer micelles. The beamlines provided high brilliance X-ray light in the energy range of 130-1500 eV. In order to obtain elemental contrast X-PEEM images (field of view 20 μm), at cobalt $L_{3,2}$-edge absorption edge and below the absorption edge were recorded sequentially by using a total electron yield (TEY) mode. The bright spots correspond to individual CoNPs, which has been confirmed by X-ray absorption spectroscopy. The base pressure in the X-PEEM was 2×10^{-8} mbar and annealing of the samples was started at this condition. Dosage of oxygen in was controlled at a X-PEEM pressure of 5×10^{-7} mbar, while that of hydrogen was 1×10^{-6} mbar. Hydrogen reduction was conducted at 623 K (SLS) or 773 K (DLS) for 1 h, while oxygen oxidation was at 573 K for 1 h, and then hydrogen was introduced again under the conditions as the first reduction. Metallic cobalt refence was recorded at DLS by using a cobalt foil. All the data were analysed by the software of ImageJ 1.52e and OriginPro 2017, and the spectra of Co L_3-edge below 776 eV were smoothed.

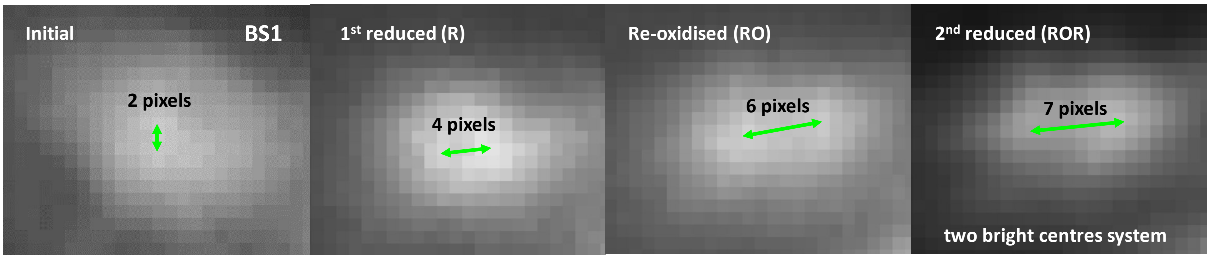

Figure S5. X-PEEM images of observed movement of CoNPs supported on $SiO_xSi(100)$ during ROR (623 K reduction) process. The field of view in the whole ROR process was maintained as 20 μm (512x512 pixels), thus each pixel in the X-PEEM image represents a same distance value (39 nm). As each NP in the X-PEEM image shows as only a bright spot, the two-bright-centre system in the X-PEEM images ($Co/SiO_xSi(100)$,) means a two-NP system there. The distance between the two bright spots become longer, meaning at least one of the NP moving away from the other one.

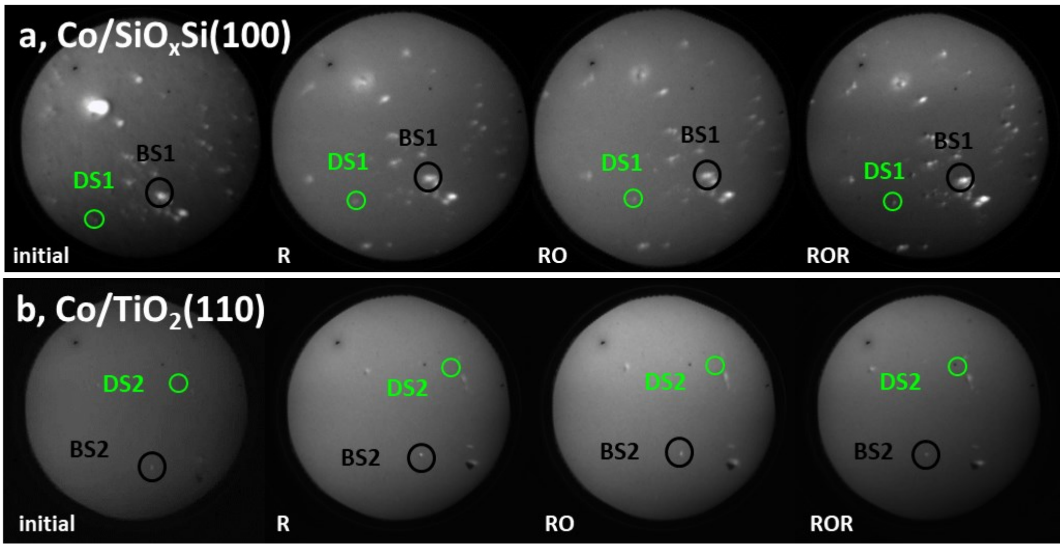

Figure S6. X-PEEM images of selected regions in the process of ROR. The bright spots distributed on both substrates during ROR in the X-PEEM images are CoNPs, with the analysed NPs circled. The spots with higher brightness (BS1, BS2) mean the bigger NPs while the lower ones (DS1, DS2) are the smaller NPs.

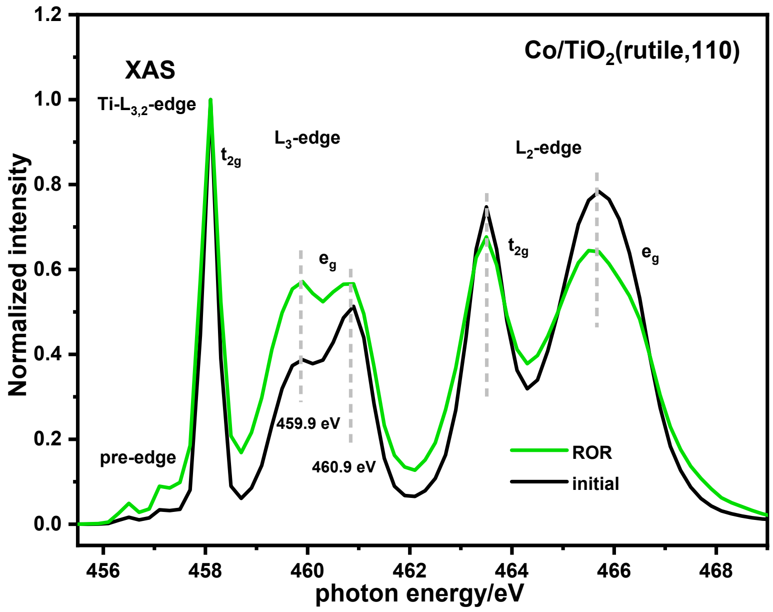

Figure S7. XAS spectra of Ti $L_{3,2}$-edge on $Co/TiO_2(110)$ before and after ROR process. Titanium in the processes is T^{4+} without any Ti^{3+} detected, as evidenced by the lack of absorption energy shift.[9] After reduction the TiO_2 crystal structure is changed from rutile to a mixture (TiO_2(II)) of anatase and rutile,[10] as confirmed by the relative height changes of peaks at 459.9 eV and 460.9 eV. These changes could be due to the low pressure and high temperature during ROR experiment or promotional effect of cobalt.

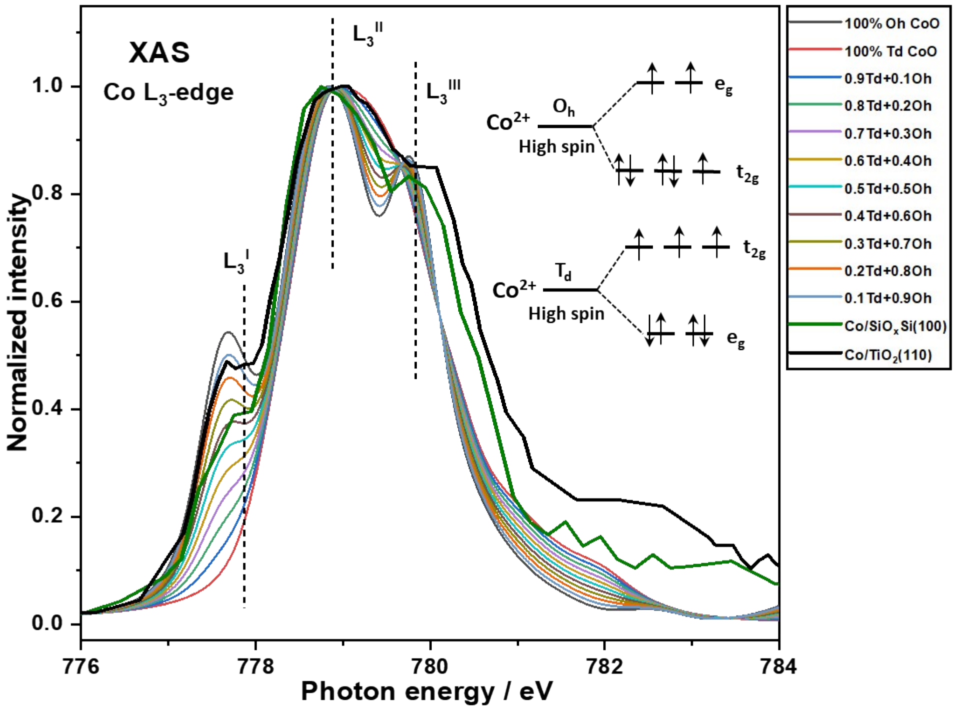

Figure S8. Comparison of Co L_3-edge XAS spectra between initial state of a CoNP on $TiO_2(110)$ and $SiO_xSi(110)$, and standard T_d, O_h Co^{2+} XAS spectra. The mixed XAS spectra of T_d and O_h Co^{2+} were calculated by linear combination, with weights denoted in the figure legend. The photon energies have been corrected to a same value. Obviously, the initial state of the CoNPs on $TiO_2(110)$ is only O_h Co^{2+}, while that on $SiO_xSi(110)$ is a mixture of T_d and O_h Co^{2+}. The corresponded electron arrangement of the cobalt in crystal field is shown as well.

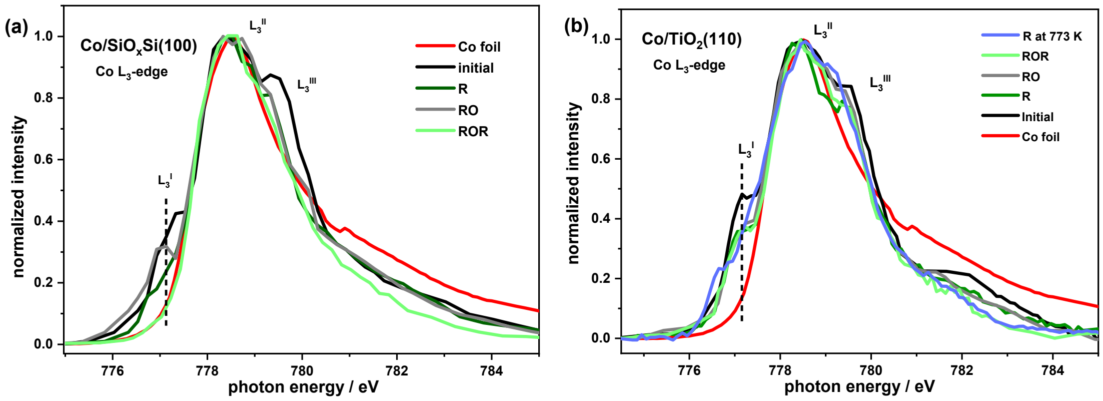

Figure S9. XAS spectra of Co L_3-edge of circled CoNPs (Figure 2e-g) supported on $SiO_xSi(100)$ (a) and $TiO_2(110)$ (b) substrate at different steps of the ROR process.

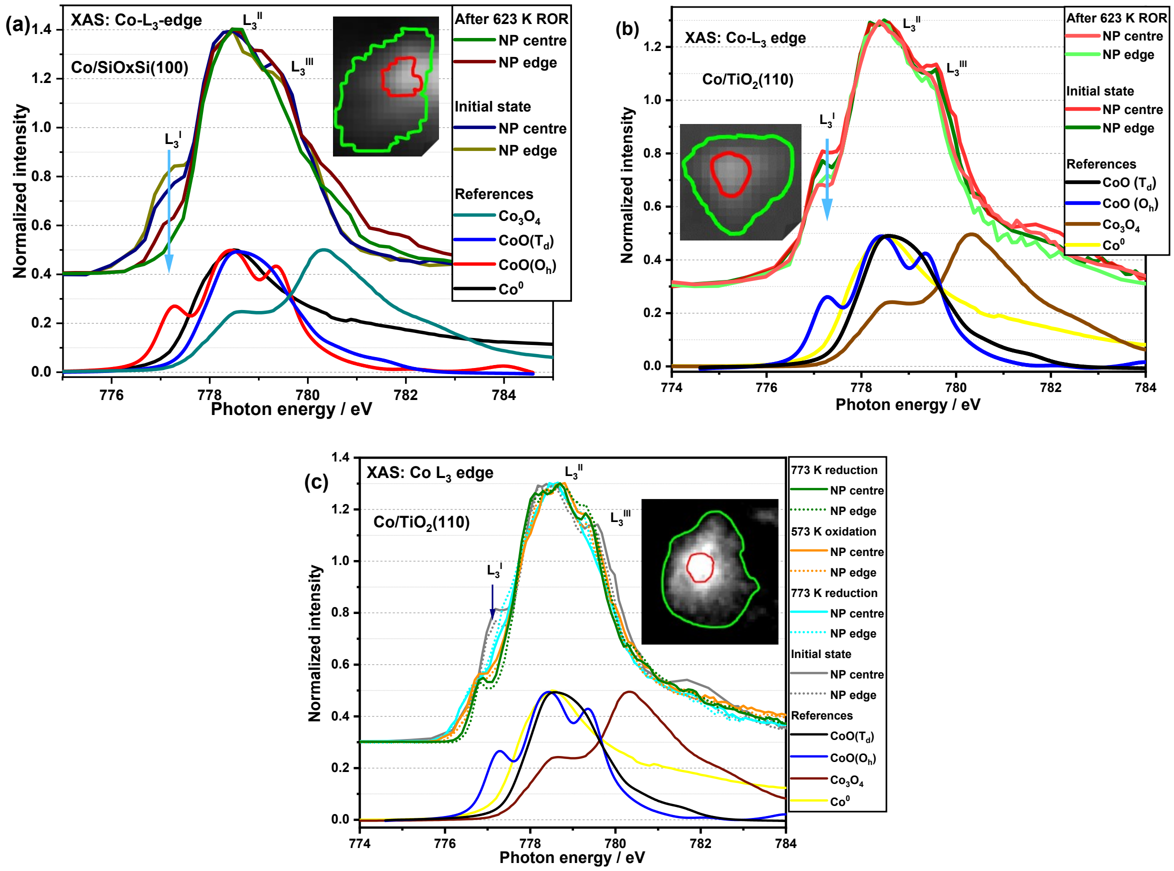

Figure S10. Comparison of XAS spectra of Co L_3-edge in the centre and edge of a CoNP in $Co/SiO_xSi(100)$ (a) and $Co/TiO_2(110)$ (b) before and after 623 K ROR treatment. (c) XAS spectra of Co L_3-edge in centre and at edge of Co NP in $Co/TiO_2(110)$ during 773 K ROR treatment. Insert is the X-PEEM screenshot with defined edge (green) and centre (red) of the NP.

6. Surface X-ray Scattering

Grazing incidence small-angle X-ray scattering (GISAXS) experiments were conducted at the SIXS beamline, SOLEIL, using flow reactor.[11] The focused beam of ca. 0.3×0.3 mm^2 with photon energy of 15 keV was directed on the sample at an incident angles of 0.2° and 0.15°. The sample to detector distance (SD) was calibrated using several di□raction orders of Ag behenate. The modulus of the scattering vector s was calculated as $q = 2\sin\theta/\lambda$, where θ is the Bragg angle and λ - the wavelength of the photons. The EIGER R 1M (1030 x 1065 pixels, 75 µm pixel size) was used for GISAXS data.

Additional GIXD experiments were performed at the I07 beamline, DLS, with a configuration similar to that previously used by Martin et al.[12]. The photon energy was 10 keV. The SD was calibrated using the shift of the direct beam position on the 2D detector mounted on the diffractometer arm moving out-of-plane. GIXD was measured using a small swing arm area detector (Pilatus 100 K, 172×172 μm^2 pixel size, 487×195 pixels).

In both experiments the reduction was performed in H_2 at flow rate of 30 mL/min at 623 K for TiO_2 and SiO_xSi supported CoNPs.

The GIXD data reduction was performed using Binoculars software package[13] and DAWN Science was used for GISAXS[14]. The 2D images were visualised in Igor Pro and 1D profiles in the OriginPro 2017.

The full 2D GISAXS images were fitted using the BornAgain v1.14 software.[15] A model consisting of Co truncated spheres supported on the flat substrate was used to represent the 2D catalysts that was also supported by microscopy data. The scattering cross-section for NPs was calculated using the Decoupling Approximation for which the position of the particles is independent of their size and the interference function is defined by an effective structural factor calculated for the average nanoparticle size. The refractive indices of Co, SiO_2 and TiO_2 were taken from the literature.[16]

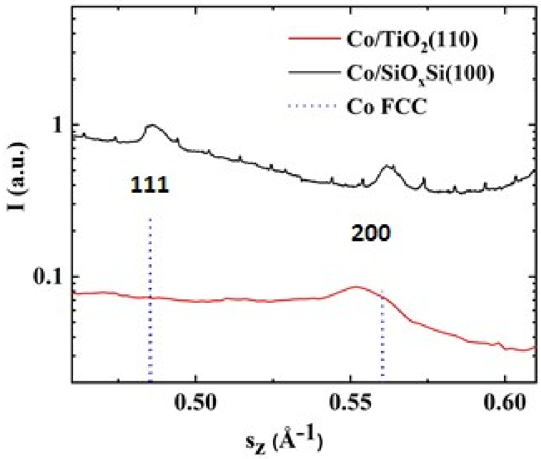

Figure S11. Comparison of 1D GIXD out-of-plane projections of the reduced Co/SiO_xSi(100) and Co/TiO_2(110) catalysts showing a difference in crystalline structure of the Co NPs on different supports. In case of silica the presence of crystalline (111) and (200) peaks corresponding to FCC phase of metallic cobalt can be observed, meanwhile the signal from the crystalline cobalt is almost absent for the titania support.

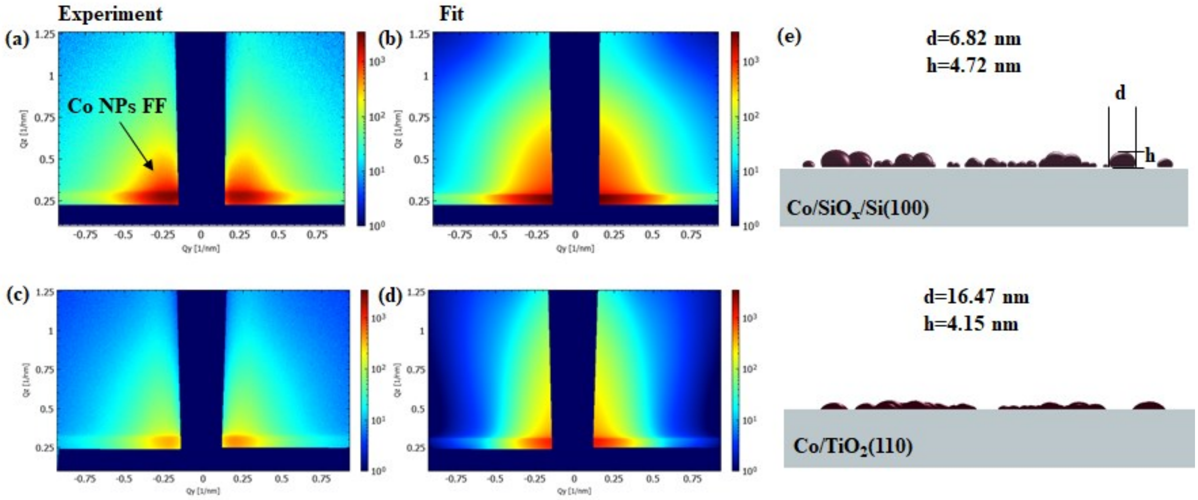

Figure S12. Experimental (a, c) vs. fitted (b, d) 2D GISAXS images of reduced Co/SiO_xSi(100) (a, b) and Co/TiO_2(110) (c, d) catalysts with visible Co nanoparticle form factor (FF). Cobalt spreading is corroborated by the larger diameter and smaller height for titania support in comparison to silica. (e) Side view of the model resulted from the fit showing the average diameter (d) and height (h) of the NPs. According to the fitting the average lateral diameter of particle increases while the height decreases for titania in comparison to silica.

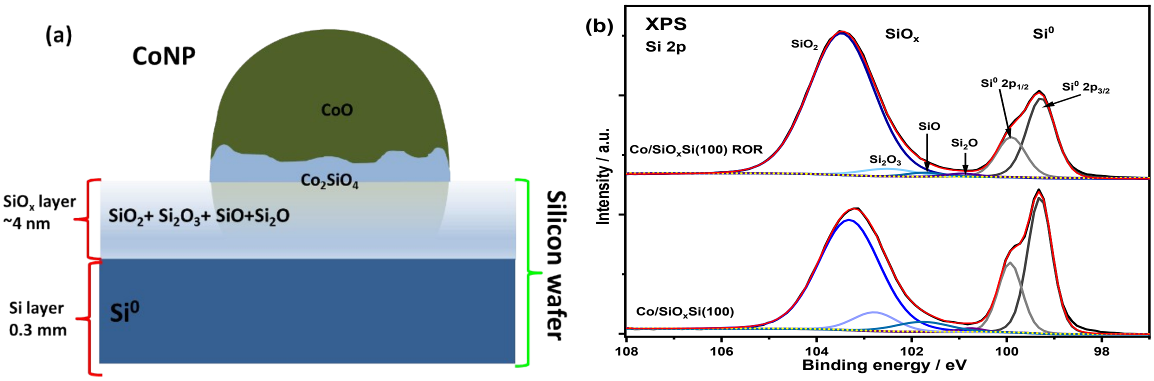

Figure S13. (a) Schematic of the SiO_x layer in Si(100) substrate and interaction of CoNPs with SiO_xSi substrate; (b) fitted XPS Si 2p spectra showing the presence of Si^{n+}($1 \leqslant n \leqslant 4$).

Note: For access to the raw data collected in this manuscript, please email the corresponding author.

References

1. Wiedwald, U.; Han, L.; Biskupek, J.; Kaiser, U.; Ziemann, P. Preparation and Characterization of Supported Magnetic Nanoparticles Prepared by Reverse Micelles. *Beilstein J. Nanotechnol.* ***2010***, 1, 24-47.

2. Boyen, H. G.; Kästle, G.; Zürn, K.; Herzog, T.; Weigl, F.; Ziemann, P.; Mayer, O.; Jerome, C.; Möller, M.; Spatz, J. P.; *et al.* A micellar Route to Ordered Arrays of Magnetic Nanoparticles: From Size-Selected Pure Cobalt Dots to Cobalt-Cobalt Oxide Core-Shell Systems. *Adv. Funct. Mater,* ***2003***, 13, 359-364.

3. Karim, W.; Kleibert, A.; Hartfelder, U.; Balan, A.; Gobrecht, J.; van Bokhoven, J. A.; Ekinci, Y. Size-Dependent Redox Behavior of Iron Observed by In-Situ Single Nanoparticle Spectro-Microscopy on Well-Defined Model Systems. *Sci. Rep.* ***2016***, 6, 18818.

4. Deese, S. M.; Englade-Franklin, L. E.; Hill, L. J.; Pyun, J.; Chan, J. Y.; Garno, J. C. Subsurface Imaging of the Cores of Polymer-Encapsulated Cobalt Nanoparticles Using Force Modulation Microscopy. *J. Phys. Chem. C* ***2017***, 121, 23498-23504.

5. Mohtasebzadeh, A. R.; Ye, L.; Crawford, T. M. Magnetic Nanoparticle Arrays Self-Assembled on Perpendicular Magnetic Recording Media. *Int. J. Mol. Sci.* ***2015***, 16, 19769-19779.

6. Liu, X.; Atwater, M.; Wang, J.; Huo, Q. Extinction Coefficient of Gold Nanoparticles with Different Sizes and Different Capping Ligands. *Colloids Surf. B* ***2007***, 58, 3-7.

7. Prathibha, V.; Karthika, S.; Cyriac, J.; Sudarasanakumar, C.; Unnikrishnan, N. V. Synthesis of Pure Anatase TiO_2 Nanocrystals in SiO_2 Host and the Determination of Crystal Planes by Image J. *Mater. Lett.* ***2011***, 65, 664-666.

8. Biesinger, M. C.; Payne, B. P.; Grosvenor, A. P.; Lau, L. W.M.; Gerson, A. R.; Smart, R. St. C. Resolving Surface Chemical States in XPS Analysis of First Row Transition Metals, Oxides and Hydroxides: Cr, Mn, Fe, Co and Ni. *Appl. Surf. Sci.* ***2011***, 257, 2717-2730.

9. Treske, U.; Heming, N.; Knupfer, M.; Büchner, B.; Gennaro, E. D.; Khare, A.; Scotti, U.; Uccio, D.; Granozio, F. M.; Krause, S.; et al. Universal Electronic Structure of Polar Oxide Hetero-Interfaces. *Sci. Rep.* ***2015***, 5, 14506.

10. Lee, Y. J.; de Jong, M. P.; van der Wiel, W. G. Electronic Structure of Co^{2+} Ions in Anatase Co:TiO_2 in Relation to Heterogeneity and Structural Defects. *Phys. Rev. B* ***2011***, 83, 134404.

11. Van Rijn, R.; Ackermann, M. D.; Balmes, O.; Dufrane, T.; Geluk, A.; Gonzalez, H.; Isern, H.; de Kuyper, E.; Petit, L.; Sole, V. A. et al. Ultrahigh Vacuum/High-Pressure Flow Reactor for Surface X-Ray Diffraction and Grazing Incidence Small Angle X-Ray Scattering Studies Close to Conditions for Industrial Catalysis. *Rev. Sci. Instrum.* ***2010***, **81**, 014101.

12. Martin, D. J.; Decarolis, D.; Odarchenko, Y. I.; Herbert, J. J.; Arnold, T. Rawle, J. Nicklin, C.; Boyenf, H. G.; Beale, A. M. Reversible Restructuring of Supported Au Nanoparticles during Butadiene Hydrogenation Revealed by Operando GISAXS/GIWAXS. *Chem. Commun.* ***2017***, 53, 5159-5162.

13. Roobol, S.; Onderwaater, W.; Drnec, J.; Felici, R.; Frenken, J. BINoculars : Data Reduction and Analysis Software for Two-Dimensional Detectors in Surface X-ray Diffraction. *J. Appl. Crystallogr.* ***2015***, 48, 1324-1329.

14. Basham, M.; Filik, J.; Wharmby, M. T.; Chang, P. C. Y.; Kassaby, B. E.; Gerring, M.; Aishima, J.; Levik, K.; Pulford, B. C. A.; Sikharulidze, I.; et al. Data Analysis WorkbeNch (DAWN). *J. Synchrotron Radiat.* ***2015***, 22, 853-858.

15. Pospelov, G.; Herck, W. V.; Burle, J.; Loaiza, J. M. C.; Durniak, C.; Fisher, J. M.; Ganeva, M.; Yurov, D.; Wuttke, J. BornAgain: Software for Simulating and Fitting Grazing-Incidence Small-Angle Scattering. *J. Appl. Crystallogr.* ***2020***, 53, 262-276.

16. Henke, B. L.; Gullikson, E. M.; Davis, J. C. X-Ray Interactions: Photoabsorption, Scattering, Transmission, and Reflection at E = 50-30,000 eV, Z = 1-92. *At. Data Nucl. Data Tables* ***1993***, 54, 181-342.
